# Pathogenic Potential of *Erysipelothrix piscisicarius* in Pigs and Its Implications for Surveillance in Brazil

**DOI:** 10.1155/tbed/5618952

**Published:** 2025-09-05

**Authors:** Fernando Moreira Petri, Giovana da Silva Nogueira, Gustavo M. R. Simão, Ana K. Panneitz, Gabriel A. de Aguiar, Ana Clara A. de Lima, Eduarda R. Braga, Kellem do Carmo, Suzana S. Kuchiishi, Adrienny T. Reis, Fabio A. Vannucci, Luís Guilherme de Oliveira

**Affiliations:** ^1^School of Agricultural and Veterinary Sciences, São Paulo State University (Unesp), Jaboticabal 14884-900, São Paulo, Brazil; ^2^Agroceres PIC Company, Rio Claro 13502-741, São Paulo, Brazil; ^3^Animal Health Diagnostic Center (CEDISA), Concórdia 89715-899, Santa Catarina, Brazil; ^4^Phibro Animal Health Corporation, Guarulhos 07112-070, São Paulo, Brazil; ^5^Department of Veterinary and Biomedical Sciences, College of Veterinary Medicine, University of Minnesota, Saint Paul 55108, Minnesota, USA

**Keywords:** emerging diseases, experimental infection, swine erysipelas

## Abstract

*Erysipelothrix* (*E*.) *piscisicarius* is an emerging pathogen previously described in fish and more recently isolated from a clinical outbreak in swine. This study aimed to evaluate the course of infection and pathological outcomes of *E. piscisicarius* in pigs using an experimental intradermal challenge model. Twenty-six 70-day-old pigs were randomly allocated into three groups: high-dose (HD group, *n* = 10) and low-dose (LD group, *n* = 10) were challenged intradermally with 10^10^ colony forming units (CFUs) and 10^8^ CFU using a Brazilian field isolate obtained from a pig with erysipelas-like lesions and confirmed by whole-genome sequencing (WGS) and average nucleotide identity (ANI), respectively, while CONT (control, *n* = 6) served as a negative control. Clinical monitoring, hematological assessments, acute-phase proteins (APPs) quantification, bacteriological culture, and quantitative PCR (qPCR) targeting the *spaC* gene were performed over 14 days. Challenged pigs developed mild clinical signs, including transient fever and characteristic rhomboid skin lesions resembling classical swine erysipelas. No mortality occurred. Hematological analysis revealed significant reductions in red blood cell (RBC) count, hemoglobin (HGB), and hematocrit (HCT), particularly in the LD group at 7 and 14 days post-challenge (dpc) (*p*  < 0.05), suggestive of inflammatory anemia. APP analysis showed a significant increase in ceruloplasmin across all groups over time, whereas transferrin levels decreased only in the control group. Bacterial isolation was unsuccessful; however, qPCR detected *E. piscisicarius* deoxyribonucleic acid (DNA) in blood, skin, liver, and spleen samples, confirming systemic dissemination, particularly at 7 dpc. These findings demonstrate that *E. piscisicarius* can induce clinical and pathological alterations in swine, although with mild severity under experimental conditions. Moreover, the study highlights the importance of differentiating *E. piscisicarius* from *E. rhusiopathiae* in diagnostics, given the potential limitations of current vaccine strategies.

## 1. Introduction

In Brazil, swine erysipelas has historically caused significant economic losses, often manifesting as acute sepsis, arthritis, reproductive issues, or rhomboid skin lesions during slaughterhouse inspections [1, 2]. While such outbreaks were traditionally attributed to *Erysipelothrix* (*E*.) *rhusiopathiae*, the recent discovery of *E. piscisicarius—* part of an emergent clade infecting fish, poultry, turkeys, and pigs [3, 4]—raises the hypothesis that this species may also be contributing to swine disease in pig farms from Brazil.

*Erysipelothrix* is a genus of family Erysipelothrichaceae, order Erysipelotrichales, class Erysipelotrichia and phylum Firmicutes [5]. Currently, 11 different species are described: *E. rhusiopathiae*, *E. tonsillarum*, *Erysipelothrix* species 1, 2, and 3, *E. inopinata*, *E. larvae*, *E. piscisicarius*, *E. anatis*, *E. aquatica*, and *E. urinaevulpis* [6, 7]. In addition, *E. amsterdamensis* has recently been proposed [8].

These bacteria are classified as Gram-positive, nonmotile, nonsporulating, nonacid-fast, facultative anaerobic, with optimal growth between 30 and 37°C. Colonies are small (0.1–0.5 mm in diameter), circular, and clear after 24 h of incubation, becoming slightly larger and rougher with irregular edges after 48 h. Most strains exhibit a narrow zone of partial hemolysis (α-hemolytic) on blood agar, often with a greenish color [5, 9].

Among them, *E. rhusiopathiae* is the most relevant to swine health, capable of causing acute, subacute, or chronic disease. Chronic lameness and arthritis are common sequelae, impacting animal growth and leading to condemnation at slaughter. The acute form often features characteristic pink, red or purple, raised, firm or square “diamond-shaped skin” that fade within 4–7 days in nonfatal cases [9]. Though *E. tonsillarum* species has also been associated with chronic arthritis and vegetative valvular endocarditis, it is frequently isolated from healthy pigs [10, 11].

Other species already described, including *E. piscisicarius* (previously designated *E*. sp. “strain 2” [4], have primarily been reported in aquatic animals. First described by Pomaranski et al. [3], *E. piscisicarius* is a pathogen in fish aquaculture, associated with orofacial ulceration, necrotizing dermatitis, and systemic dissemination. It can colonize mucosal surfaces of teleost fish without causing clinical signs, but once disease manifests, it is associated with necrotizing dermatitis, myositis, and extensive bacterial proliferation [12]. In experimental infections of fishes, high mortality was observed within the first 2 weeks due to bacterial toxicity during tissue colonization. Early infection signs include multifocal cutaneous ulceration and epithelial necrosis, with mild neutrophil, lymphocyte, and macrophage infiltrates in the dermis. These lesions may worsen over time [13]. Also, in 2022, a human case was reported in which *E. piscisicarius* was isolated after a shrimp-handling lesion [14].

Most recently, following an outbreak of erysipelas-like disease in a Brazilian swine farm in 2022, *E. piscisicarius* was isolated and confirmed by whole-genome sequencing (WGS). Therefore, this study aimed to characterize the clinical and pathological effects of this species in healthy pigs experimentally challenged with the field isolate.

## 2. Materials and Methods

### 2.1. Experimental Design and Animal Challenge

Twenty-six 70-day-old male pigs originating from a commercial high-health-status farm were used in this study. The farm of origin maintained a routine vaccination program against *E. rhusiopathiae*, using the *Eriseng Parvo Lepto* (HIPRA, Spain), a commercial inactivated vaccine administered to replacement gilts at 175 and 195 days of age and to lactating sows between 7 and 10 days after farrowing. Additional information is available in Table S1. Although pigs were negative for *E*. spp. by quantitative PCR (qPCR) at the time of challenge, no serological testing was performed to assess maternally derived antibodies (MDAs). The animals were housed at the facilities of the Swine Medicine Laboratory (Unesp/FCAV Jaboticabal, São Paulo, Brazil). The animals underwent a 4-day acclimation period during which they received water ad libitum and antibiotic-free feed appropriate for the corresponding life stage. After the acclimation period, the pigs were weighed and identified with individuals ear tags. Three experimental groups were established as follows: high-dose (HD group), 10 pigs challenged at 73 days of age with 10^10^ colony forming units (CFUs) of *E. piscicicarius* per pig; low-dose (LD group), 10 pigs challenged at 73 days of age with 10^8^ CFU of *E. piscicicarius* per pig; control (CONT group), six nonchallenged pigs. All animals were negative for *E. rhusiopathiae* and *E. piscicicarius*, as confirmed by qPCR in whole blood and tonsil swab [15, 16].

The bacterial challenge was conducted using a field isolate obtained from a clinically affected pig showing erysipelas-like clinical lesions on a commercial Brazilian swine farm. Notably, the herd was routinely vaccinated against *E. rhusiopathiae*, yet clinical disease still occurred. Moreover, the property also practiced fish farming, raising the possibility of interspecies transmission or environmental persistence of *E*. spp.

Initial identification was based on colony morphology and Gram staining. Species-level confirmation was achieved by WGS, performed at the Veterinary Diagnostic Laboratory (University of Minnesota, USA). The WGS data showed high similarity to the *E. piscisicarius* 15TAL0474 (Figures S1 and S2). Moreover, average nucleotide identity (ANI) between our isolate and the type strain *E. piscisicarius* 15TAL0474 (GenBank accession NZ_CP034234.1) was ≥99.1% (Table S2), exceeding the ≥95% species-level threshold. ANI was calculated using the EZBioCloud platform tool [17]. Additionally, phylogenomic analysis based on WGS (TYGS platform) [18] confirmed that the isolate tightly with *E. piscisicarius*, and clearly apart from *E. rhusiopathiae* and other *E*. spp. (Figure S3). To our knowledge, no previous study has experimentally characterized *E. piscisicarius* infection in pigs.

For challenge, each pig in groups HD and LD was inoculated intradermally in both sides of the dorsal region with 0.2 mL of bacterial suspension in brain heart infusion (BHI) broth, containing either 10^10^ CFU (HD) and 10^8^ CFU (LD) of the isolated field strain [19]. Prior to injection, the inoculation site was shaved to ensure proper penetration and lesion monitoring. CONT group received no bacterial inoculation on D0 (Day 0 of the study).

The experimental trial involving pigs adhered to ethical standards and guidelines for animal experimentation by the Brazilian College of Experimentation. All procedures were approved by the Animal Ethics Committee of School of Agricultural and Veterinary Sciences, São Paulo State University, as registered by Protocol Number #006313/23 and regularly checked by veterinarians.

### 2.2. Clinical Evaluation and Blood Sample Collection

After the challenge at D0, rectal temperature, clinical signs and skin lesions were monitored daily in all animals. Blood sampling was performed at D2, D7, and D14 via jugular puncture using closed vacuum system (Vacutainer, BD, USA).

For hematological and molecular analysis, blood was collected into sterile tubes containing ethylenediaminetetraacetic acid (EDTA). Immediately after hematological analysis using the whole blood sample, aliquots were transferred in duplicate to DNAse- and RNAse-free microtubes (Kasvi, Brazil) and stored at −80°C until further qPCR processing.

Additionally, blood for acute-phase proteins (APPs) analysis was collected into sterile tubes containing clot activators. After clot formation, samples were centrifuged, and serum was aliquoted into sterile microtubes and stored at −20°C until further use.

### 2.3. Necropsy and Sample Collection

Seven (D7) and 14 (D14) days post-challenge (dpc), half of pigs randomly in each group were euthanized through intramuscular administration of a combination of acepromazine, ketamine and xylazine (0.05, 10, and 0.05 mg/kg, respectively). Euthanasia occurred with a puncture of 10 mL of lidocaine 2% in the atlanto-occipital space, approved by the Federal Council of Veterinary Medicine, Brazil. Following evisceration, the organs were assessed according to macroscopic alterations. Liver and spleen of all animals were weighed aiming at the evaluation of size alterations in the organs from challenged and nonchallenged pigs.

Samples of skin, heart, lungs, liver, spleen, lymph nodes, kidney, brain, ileum, cecum, and testicle were collected aseptically, placed into sterile plastic bags (Whirl-Pak, Madison, WI, USA), also, swabs from blood and joint were collected. All samples were kept under refrigeration until processing intending bacterial analysis. Also, tissue fragments were collected in triplicates of 1 cm × 2 cm each for qPCR detection and microscopic lesions assessment. Two of the triplicates were immediately transferred into DNAse- and RNAse-free microtubes (Kasvi, Brazil) and stored at −80°C until qPCR further processing, and the third one was fixed in 10% (v/v) buffered formalin for histopathological analysis.

### 2.4. Average Daily Weight Gain (ADWG) Assessment

The ADWG was calculated by subtracting the initial weight of the animal in the challenge (D0) from the final weight (euthanasia day), and the result was divided by the number of days between challenge day and the euthanasia of the animal.

### 2.5. Laboratory Analysis

#### 2.5.1. Blood Cells Count

Blood samples collected with EDTA anticoagulant were submitted to red blood cell (RBC) count, white blood cell counts (WBC) and platelet (PLT) count, hemoglobin (HGB) and hematocrit (HCT) content, and hematimetric indexes, including mean corpuscular volume (MCV), mean corpuscular hemoglobin (MCH), and mean corpuscular hemoglobin concentration (MCHC). Hematological analyses were performed in an automatic device (pocH-100 iV Diff, Sysmex Inc., Kobe, Japan), following the manufacturer's instructions.

#### 2.5.2. Bacteriological Analysis

For bacteriology analysis, tissue samples (skin, heart, lungs, liver, spleen, lymph nodes, kidney, brain, ileum, cecum, and testicle), as well as swabs from blood and joints aseptically collected at D7 and D14, were processed and streaked onto blood agar (5% sheep blood, Oxoid, Hampshire, UK) and MacConkey agar (Oxoid, Hampshire, UK). Plates were incubated aerobically at 36 ± 1°C for 24–48 h, and bacterial identification was performed according to Whitman, [5]. Briefly, *E. rhusiopathiae* typically appears as pleomorphic rods and produces hydrogen sulfide (H_2_S) in triple sugar iron (TSI) and sulfide-indole-motility (SIM) media. In contrast, *E. piscisicarius* displays a coccoid rod morphology and lacks H_2_S production in both assays [13].

#### 2.5.3. DNA Extraction From Biological Samples

The tissue and blood samples were extracted using the in-house Tris-HCl protocol [20]. For skin, liver, and spleen samples, 30 mg of tissue were used, and 250 µL for blood samples. Measurement of the DNA concentration of the samples was performed with spectrophotometry (NanoDrop One, Thermofisher Scientific, USA), having as an exclusion factor the samples that did not reach the purity of 1.8–2.0 ng/µL DNA in the 260/280 nm ratio.

#### 2.5.4. qPCR Assays Targeting the *spaC* Gene From *E. piscisicarius*

All extracted DNA samples were submitted to qPCR in duplicates assay aiming at the detection of *E. piscisicarius* using published primers and established protocols for identification of gene of surface protective antigen *spaC* type [16]. As a positive control, DNA extracted from bacterial culture of *E. piscisicarius* 15TAL0474, were utilized in the assays. All samples were subjected to qPCR for *E. rhusiopathiae* [15].

#### 2.5.5. Sodium Dodecyl Sulfate-Polyacrylamide Gel Electrophoresis (SDS–PAGE) Analysis of Acute-Phase Serum Proteins

Serum samples were diluted at a ratio of 1:3 (10 µL of serum and 30 µL of phosphate-buffered saline [PBS 1x, pH 7.4]; Sigma–Aldrich, St. Louis, MO, USA) prior to electrophoresis. Protein fractionation was performed using SDS–PAGE, following the method described by Laemmli [21]. After electrophoresis, gels were stained for 10 min with 0.25% (w/v) coomassie brilliant blue solution and, subsequently, bleached in a 7% acetic acid until protein bands were clearly visible. Protein concentrations were quantified by densitometry using a computerized scanner (Shimadzu CS-9301 PC, Tokyo, Japan). A molecular weight (MW) marker (Sigma–Aldrich, St. Louis, MO, USA) was used as a standard reference, along with purified porcine proteins for identification and confirmation of MW, including albumin, transferrin, haptoglobin, ceruloplasmin, α1-antitrypsin, α1-acid glycoprotein, IgA, and IgG (Sigma–Aldrich, St. Louis, MO, USA).

The total serum protein concentration was determined by the biuret method using a semiautomated spectrophotometer (Labquest, Labtest Diagnóstica, Lagoa Santa, MG, Brazil) and commercial reagent kits (Labtest Diagnóstica, Lagoa Santa, MG, Brazil).

### 2.6. Data Analysis

The variables within each group and time point were assessed for normality and homoscedasticity using the Shapiro–Wilk and Bartlett tests, respectively. Mean differences were determined by Tukey's test (*p* < 0.05). Variables not meeting the assumptions underwent the nonparametric Kruskal–Wallis test (*p* < 0.05), and in cases of significance, the Dunn test (post-hoc) was employed. To investigate potential associations between bacterial burden and host response, we performed Spearman's rank correlation analyses between qPCR Ct values obtained from tissues samples and physiological variables. Correlations were tested against final body weight, ADWG, and hematological parameters including RBC, WBC, HCT, and HGB. All graphs were plotted using GraphPad Prism 10 software (La Jolla, CA-USA).

## 3. Results

### 3.1. Clinical, Zootechnical, and Performance Parameters

**Figure 1:**
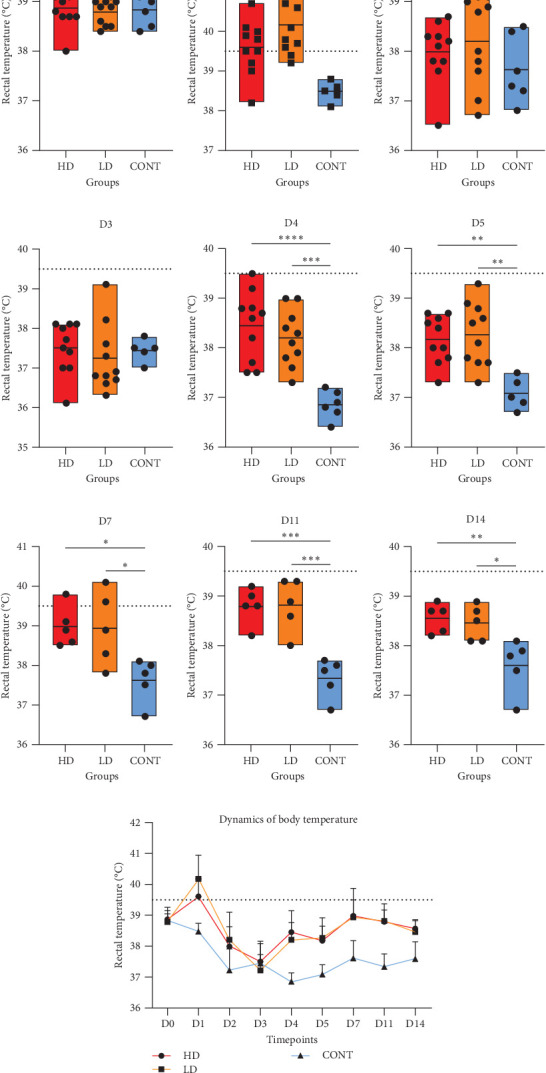
Rectal temperature measurements in pigs challenged with *E. piscisicarius*. (A–I) Individual temperatures recorded daily from D0 to D14 for each group: HD, LD, and CONT. Black bars indicate group medians. The dotted horizontal line indicates the fever threshold (39.5°C) by Dewey and Straw [22]. Asterisks indicate significant differences between groups using Tukey test (*⁣*^*∗*^*p* < 0.05; *⁣*^*∗∗*^*p* < 0.01; *⁣*^*∗∗∗*^*p* < 0.001; *⁣*^*∗∗∗∗*^*p* < 0.0001). (J) Temporal evolution of group mean ± standard error of mean.

No animals died during the experimental period. Regarding the rectal temperature of animals, 24 h post-challenge on D1, pigs from groups HD and LD showed a difference in mean temperature with the CONT group. From HD group 5/10 pigs (50%) presented fever (≥39.5°C) at this moment and 8/10 pigs (80%) from LD group. Additionally, on days 4, 5, 11, and 14 we observed statistical differences between both intradermally challenged and CONT group (Figure 1). Mean and higher and lower values of rectal temperature can be seen in Table S3.

**Figure 2:**
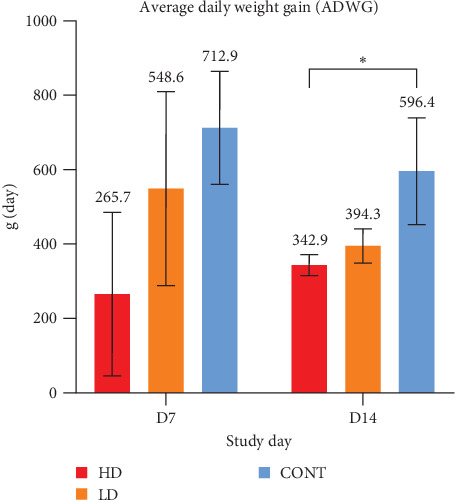
Average daily weight gain (mean ± standard error of mean) in pigs from challenged (HD and LD) and control (CONT) groups at D7 and D14. Statistical comparisons were performed using Student's *t*-test (*⁣*^*∗*^*p*  < 0.05).

At D14, pigs in the HD group exhibited significantly lower ADWG compared to the nonchallenged CONT group (343.1± 62.4 g vs. 596.4 ± 288.8 g; *p*=0.032). Although pigs in the LD group had a numerically lower gain (394.3 ± 104 g) than nonchallenged pigs, the difference was not statistically significant (*p*=0.111). No significant difference was observed between HD and LD groups (*p*=0.548; Figure 2).

### 3.2. Macroscopic and Microscopic Lesions

During the experimental period, the animals developed skin lesions following intradermal inoculation with the *E. piscisicarius* isolated strain. At 3 dpc (D3), pigs in group LD exhibited localized erythema lesions in the rostral region (4/10). On D4, pigs in group HD began to show erythema lesions (3/10), while those in group LD experienced an increase in the number of erythema lesions (5/10), both in the rostral region. Additionally, group LD pigs developed disseminated erythema lesions on their backs (5/10).

**Figure 3:**
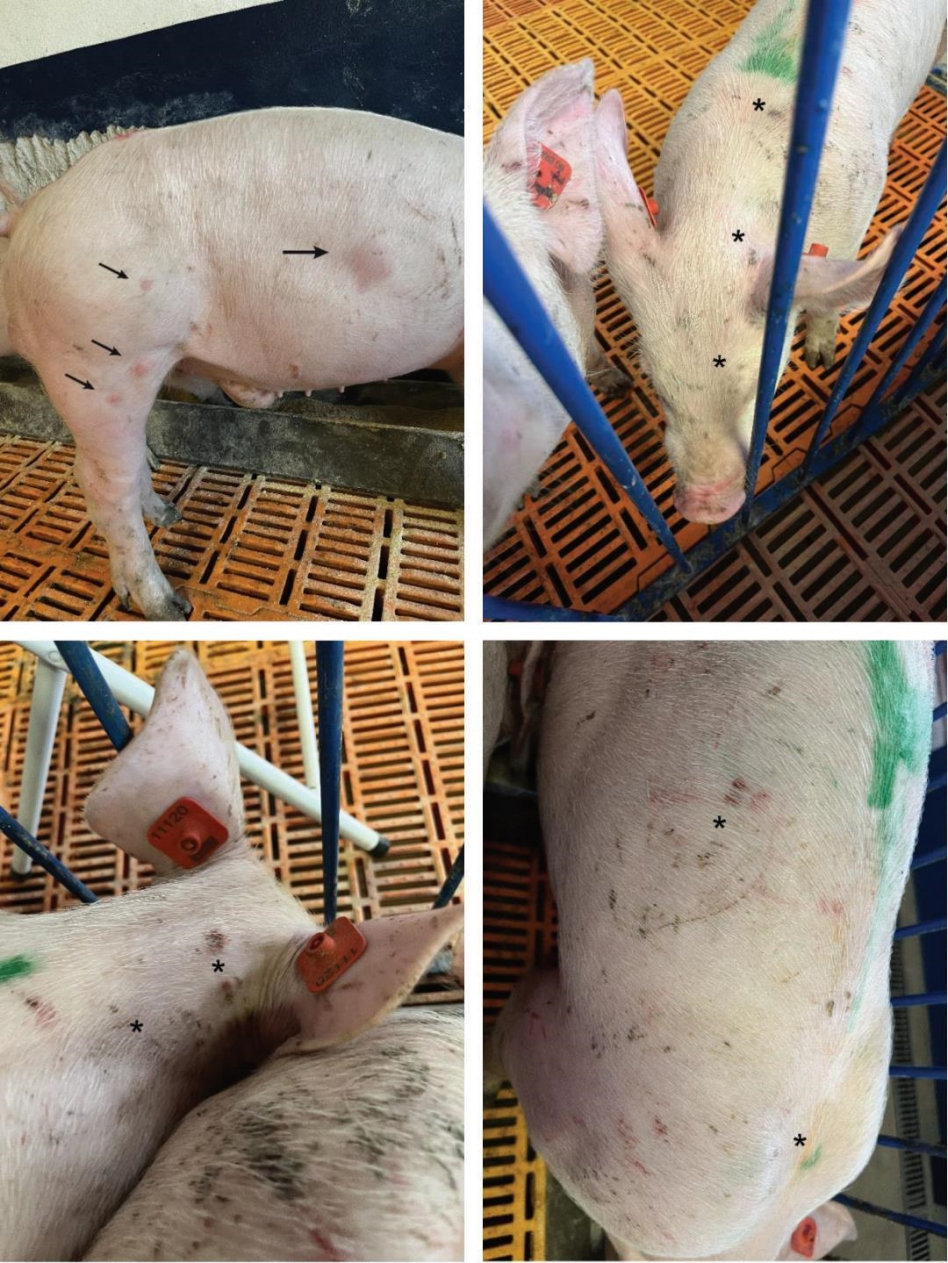
Macroscopic lesions observed in pigs challenged with *E. piscisicarius*. Arrows indicate rhomboid skin lesions, and asterisks indicate areas of erythema observed 2 days postintradermal inoculation.

Regarding rhomboid lesions, pigs in HD group (2/10) and LD group (2/10) began to develop mild lesions in the dorsal and rib regions on D4. Five days postinoculation (D5), both HD and LD showed localized erythema lesions (2/10 and 4/10, respectively) and disseminated lesions (7/10 and 4/10, respectively). Finally, rhomboid lesions were observed in 3/10 and 4/10 animals in challenged HD and LD groups, respectively. Representative images of the lesions can be seen in Figure 3.

After evisceration, all livers and spleens were weighed and evaluated macroscopically (Table 1). The animals did not present any significant macroscopic lesions, specially associated with erysipelas-disease, like adherence or splenomegaly and hepatomegaly. In addition, a significant positive correlation was observed between body weight and liver weight within both challenged groups. In the HD group, body weight was strongly correlated with liver weight (Spearman's rho = 0.782, *p*=0.011), and a similar pattern was found in the LD group (rho = 0.709, *p*=0.027). These results suggest that liver size remained proportionate to the overall growth performance of the animals, indicating that *E. piscisicarius* infection did not cause organ hypertrophy or atrophy under the conditions evaluated.

**Figure 4:**
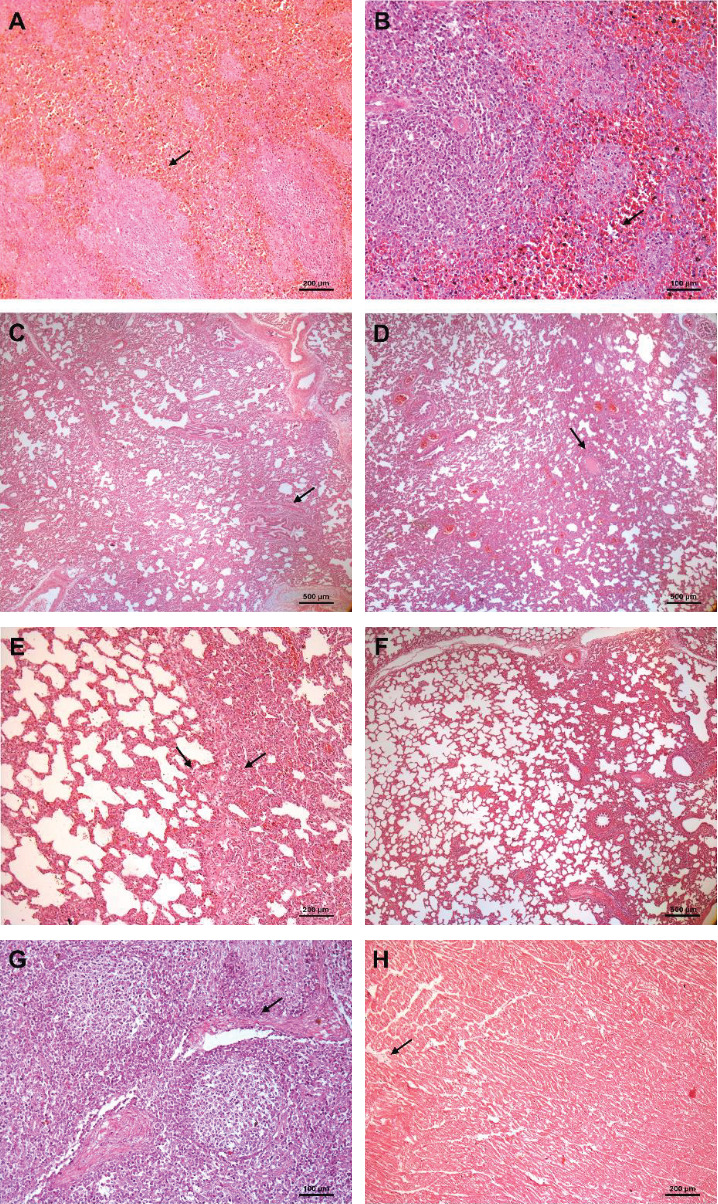
Representative histopathological findings in pigs challenged with *E. piscisicarius* (H&E stain). (A) Spleen (100x): moderate congestion of the red pulp (→); (B) spleen (200x): marked pulp congestion and mild inflammatory infiltration (→); (C) lung (40x): mild fibrinous pneumonia with alveolar exudate (→); (D) lung (40x): interstitial pneumonia with alveolar septal thickening (→); (E) lung (100x): transition between inflamed and preserved areas with alveolar septal thickening; (F) lung (40x): preserved alveolar architecture; (G) tonsil (200x): crypt dilation (→) with necrotic debris and lymphoplasmacytic infiltrate; (H) heart (100x): mild perivascular mononuclear infiltrate (→) suggestive of early pericarditis.

Histopathological evaluation revealed distinct patterns of tissue alterations among groups and timepoints (Figure 4). In the HD group, pulmonary lesions characterized by mononuclear infiltrate and pleural fibrin were consistently observed at both 7 and 14 dpc in 60% of pigs. Splenic congestion was frequent, affecting 80% of HD pigs at 7 dpc and 100% at 14 dpc. Mild pericarditis and testicular inflammation were observed only in one animal at 7 dpc. In the LD group, pulmonary lesions were present in all animals at 7 dpc (100%) but decreased prevalence to 20% at 14 dpc. Splenic congestion was minimal (2/5 at D14), while renal mononuclear infiltrates increased from 40% to 60% between 7 and 14 dpc. Mild tonsillar crypt dilation appeared in one animal at 14 dpc. Notably, dermal perivascular infiltrates were more frequent in LD, observed in 60% of pigs at D7 and 20% at D14.

In contrast, CONT group exhibited only background-level findings. No pulmonary, splenic, cardiac, or testicular lesions were detected. Renal mononuclear infiltrates and mild dermal infiltration were observed in a few individuals, as commonly reported in nonchallenged animals. Overall, the lesions identified in challenged pigs were mild but consistent with systemic and localized inflammatory responses, particularly in the lungs, spleen, and skin. Their absence or low incidence in nonchallenged pigs support their association with the experimental infection (Table 2).

### 3.3. Molecular Detection of *spaC* Gene of *E. piscisicarius* in Tissues and Blood

All tissues and blood processed for bacterial culture showed no growth suggestive of *E. spp*. Nonetheless, all samples were subjected to qPCR targeting the *spaC* gene to specifically detect *E. piscisicarius*, as well as a separate qPCR targeting *E. rhusiopathiae*. All samples from CONT group were negative for both assays. Additionally, *E. rhusiopathiae* DNA was not detected in any sample across challenged groups.

In contrast, qPCR successfully detected *E. piscisicarius* DNA in selected samples from challenged animals. At D2, *E. piscisicarius* was detected in whole blood samples of 20% of HD pigs (mean cycle threshold [Ct] = 35.7 ± 1.1) and 40% of LD pigs (Ct = 38.5 ± 0.84). At D7, the pathogen was detected in the skin of 60% of pigs in both HD and LD groups (Ct = 37.8 ± 1.5 and 37.1 ± 0.46, respectively). Liver samples were positive in 60% (HD) and 100% (LD) of animals, with Ct values of 37.5 ± 0.48 and 36.3 ± 1.06, respectively. Spleen samples yielded 100% positivity in both groups (HD: Ct = 33.4 ± 0.25; LD: Ct = 33.2 ± 0.38).

**Figure 5:**
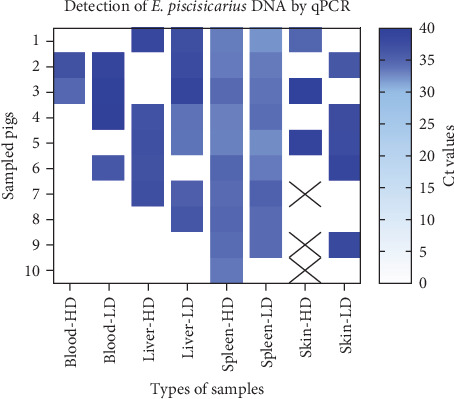
Heatmap showing cycle threshold (Ct) values from qPCR targeting the *spaC* gene in blood, liver, spleen, and skin of challenged pigs (HD and LD groups). Blue-white gradient: darker blue represents higher qPCR Ct value (lower bacterial load). White: negative by PCR. Upward diagonal pattern: not collected or missing sample prior to qPCR testing.

By D14, *E. piscisicarius* DNA was not detected in blood samples. However, the pathogen remained detectable in the skin of 40% of LD pigs (Ct = 38.4 ± 0.20), in the liver of 40% of HD and LD pigs (Ct = 37.1 ± 0.25 and 35.8 ± 0.45, respectively), and in the spleen of 100% of HD and 80% of LD animals (both Ct = 34.3 ± 0.2) (Figure 5, Table 3).

Additionally, significant negative correlations were found between qPCR Ct values in liver and spleen and key performance indicators, including body weight and ADWG. In the HD group, lower Ct values in the liver (indicating higher bacterial load) were strongly associated with reduced final body weight (*r* = –1.0, *p*=0.017). Similar trends were observed in the LD group, where spleen Ct values negatively correlated with ADWG (*r* = –0.854, *p*=0.005) (Figure S4A, B).

### 3.4. Hematological Alterations Following Challenge With *E. piscisicarius*

**Figure 6:**
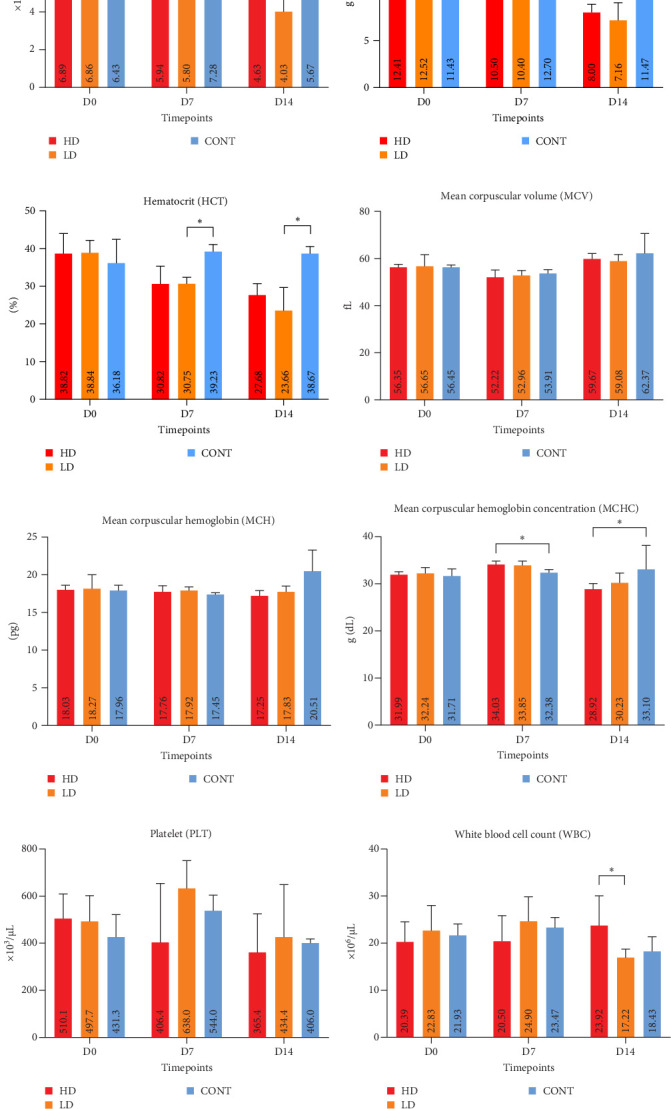
Hematological parameters in pigs after challenge with *E. piscisicarius*. (A–C) Erythrogram variables (RBC, HGB, and HCT); (D–F) Erythrocyte indices (MCV, MCH, and MCHC); (G) Platelet counts; (H) White blood cell counts. Data are presented as means (± standard error of mean). Asterisks indicate significant differences (*⁣*^*∗*^*p* < 0.05).

Peripheral blood cell responses revealed significant alterations primarily in RBC parameters and HCT following infection (Figure 6). At 7 dpc, the HD group showed a significant reduction in RBC compared to nonchallenged CONT group (*p*  < 0.05), accompanied by decreased HCT in both challenged groups (*p*  < 0.05). These alterations persisted at 14 dpc, with the LD group showing continued reductions in HCT and MCHC (*p*  < 0.05). HGB levels were also reduced at D14 in challenged groups. Notably, MCHC values were significantly lower in the HD and LD groups at both D7 and D14, suggesting the development of hypochromic anemia.

PLT counts and erythrocyte indices (MCV and MCH) remained stable across groups and timepoints. A transient increase in WBC was observed in the LD group at 7 dpc compared to CONT group (*p*  < 0.05), indicating mild leukocytosis. Additionally, in the LD group, liver Ct qPCR values were correlated with WBC (*r* = −0.821, *p*=0.034), where higher bacterial load correlates with increased WBC (Figure S4C). All hematological parameters are shown in Table S4.

### 3.5. Modulation of Serum APPs in Response to *E. piscisicarius* Challenge

The SDS–PAGE technique enabled the detection of 20 proteins, whose MWs ranged from 22 to 306 kDa. Of these, transferrin (77 kDa), ceruloplasmin (113 kDa), haptoglobin (43 kDa), albumin (667 kDa), α1-antitrypsin (557 kDa), α1-acid glycoprotein (38 kDa), nominally unidentified protein of MW 23 kDa, and immunoglobulins IgG (150 kDa) and IgA (160 kDa) were evaluated for their diagnostic importance. The results of total proteins concentrations and the protein fractions analyzed are shown in Table 4.

Among the 10 APP and immunoglobulins studied, significant changes were observed mainly for transferrin, IgG, and albumin. In the HD group, significant reduction in serum transferrin concentrations were observed at D7, while the CONT group exhibited an increase (*p*  < 0.05). A gradual increase in IgG levels was noted in both challenged groups postinfection, whereas the CONT group showed no significant changes. Additionally, serum albumin concentrations decreased on D14 in all groups, particularly in the HD group. No significant differences were observed in the serum concentrations of α1-acid glycoprotein, haptoglobin and MW 23,000.

## 4. Discussion

This study is, to our knowledge, the first to experimentally characterize infection with *E. piscisicarius* in swine. Previously associated with aquatic species, this bacterium demonstrated pathogenic potential in pigs under experimental conditions, providing valuable insights into its clinical, hematological, and pathological behavior. Fever was observed in both challenged groups (HD and LD) the day postinfection, supporting the hypothesis of a systemic inflammatory response. Although no mortality occurred, febrile responses and the presence of skin lesions indicate that *E. piscisicarius* can induce clinical manifestations in pigs, with patterns like classical erysipelas caused by *E. rhusiopathiae*, impacting the performance of affected pigs.

Hematological alterations, especially in low bacterial dose group, revealed significant reductions in RBC, HGB concentration, and HCT levels by day 7 postchallenge. These findings suggest that systemic inflammation and splenic sequestration of RBCs may have occurred, a mechanism consistent with inflammatory anemia. Cutaneous lesions, including localized and disseminated erythema, as well as rhomboid-shaped marks, developed early during infection and were visible in both challenged groups. These findings are clinically relevant, given that such lesions are characteristic of swine erysipelas and are important diagnostic indicators in field conditions. On the other hand, histopathological evaluation revealed mild but consistent pulmonary and splenic alterations in challenged animals, further supporting systemic involvement.

In addition to clinical signs and inflammatory responses, clear differences in growth performance were observed among groups. By day 14 postinfection, pigs in the HD group showed a marked reduction in average weight gain, with 342.9 g/day and LD group 394.3 g/day, while the nonchallenged group presented 596.4 g/day, reflecting a reduction in 42.5% and 33.9% in ADGW when compared to control, respectively.

Although bacterial cultures yielded no growth of *E*. spp., qPCR successfully detected *E. piscisicarius* DNA in blood and tissues. Notably, at 7 days postinfection, 100% of spleen samples and 80% of liver samples tested positive, confirming systemic dissemination. The high Ct values (mostly >33) suggest low bacterial loads, which could reflect partial immune control or lower virulence of the strain used in the challenge. Moreover, significant correlations between qPCR Ct values and performance or hematological variables were found in our study, reinforcing its biological relevance of bacterial load, even in the absence of sever disease. Also, differentiating *E. piscisicarius* from *E. rhusiopathiae* is essential for appropriate diagnosis, treatment, and disease management, especially as conventional diagnostics may not distinguish between these species.

Serum proteins modulation observed during experimental period supports a mild acute-phase response after challenge. The gradual increase in IgG levels in challenged groups may suggest activation of the humoral immune response, despite the lack of antigen-specific enzyme-linked immunosorbent assay (ELISA) for this species. Interestingly, all groups showed similar baseline IgG levels, possibly reflecting passive immunity from vaccinated sows, but only the challenged groups demonstrated progressive increases postinfection. The decrease in albumin at 14 dpc in HD and LD groups aligns with classical patterns of inflammation, where negative APPs are downregulated. Transferrin fluctuation further indicates systemic involvement, while the lack of significant changes in other proteins such as haptoglobin or α1-antitrypsin may reflect the nonestablishment of an acute infection or the early timing of sample collection relative to peak response.

Another relevant consideration emerging from our findings concerns the potential role of water as an epidemiological bridge for *E. piscisicarius* transmission between aquatic and terrestrial production systems. Since this bacterium was originally isolated from fish and is a well-recognized pathogen in aquaculture [3], its ability to cause disease in pigs raises the hypothesis of water-mediated cross-species transmission. Contaminated water sources, particularly in farms where fish farming and swine production coexist or share infrastructure, could represent a plausible route for *E. piscisicarius* into terrestrial livestock environments. Although rare, a human infection by *E. piscisicarius* has been reported [14], suggesting a potential, yet undefined, occupational risk for farm workers in direct contact with infected animals or contaminated materials.

It remains uncertain whether other *E. piscisicarius* strains exhibit similar pathogenicity, given the genetic variability previously reported among isolates from fish, poultry, and pigs [4]. Although chronic lesions were not observed during the 14-day experiment, longer studies are needed to evaluate whether *E. piscisicarius* could induce chronic manifestations such as arthritis, endocarditis, or reproductive disorders, like those seen with chronic erysipelas infection caused by *E. rhusiopathiae*. Although piglets were negative by qPCR prior to intradermal challenge, they originated from vaccinated sows, which may have conferred MDA against *E*. spp. Such passive immunity could have influenced the clinical presentation and lesion severity observed, possibly contributing to the generally mild disease course.

Interestingly, the isolate used in this study was obtained from clinical cases of erysipelas-like disease in pigs from a high-health-status herd routinely vaccinated against *E. rhusiopathiae*. Despite vaccination, affected animals developed rhomboid skin lesions and splenic congestion, from which *E. piscisicarius* was isolated and subsequently confirmed by WGS. This observation suggests that current vaccine formulations based on *E. rhusiopathiae* may offer limited cross-protection against genetically distinct species such as *E. piscisicarius*. Moreover, MDA against *E. rhusiopathiae* can persist for up to 8 weeks and have been shown to interfere with the effectiveness of vaccination [23], potentially limiting both humoral and cell-mediated immune responses in young piglets [24]. While T-cell responses were observed in 100% of piglets vaccinated at 8 and 10 weeks of age, no such responses were seen at 6 weeks in MDA-positive animals, and the humoral immunity was short [23]. These findings suggest that, even when animals are properly vaccinated against *E. rhusiopathiae*, residual MDA—depending on age and vaccine formulation—may contribute to suboptimal immune priming and susceptibility to infection. Although some antigen formulations, such as those based on NaOH-extracted proteins, may partially overcome this limitation [25], our findings reinforce that current protocols may not offer broad protection against genetically divergent species such as *E. piscisicarius*.

In addition, recent findings by Sanchez-Tarifa et al. [26] reinforce the role of sow vaccination timing in determining the level and duration of MDA against *E. rhusiopathiae* in piglets. Their study demonstrated that prefarrowing vaccination protocols led to higher and more sustained antibody titers in both sows and their offspring compared to postfarrowing immunization. Interestingly, although sows in the source herd of our study were vaccinated as replacement gilts and again 10 days postfarrowing, these recent findings suggest that postpartum vaccination may be less effective in maximizing MDA compared to prefarrowing protocols. The ability of this pathogen *E. piscisicarius* to induce clinical disease in previously vaccinated animals underscores the importance of genomic surveillance and expanded immunological targets in future vaccine strategies.

The emergence of *E. piscisicarius* reveals important gaps in current preventive and diagnostic approaches. Commercial vaccines based on *E. rhusiopathiae* may offer limited cross-protection, and standard diagnostic tests do not reliably differentiate between *Erysipelothrix* species. In cases of farms experiencing erysipelas-like outbreaks occurring despite vaccination, infection by *E. piscisicarius* should be considered. Thus, the development of species-specific qPCR and serological assays would enhance diagnostic accuracy, surveillance, and disease management.

## 5. Conclusion

In conclusion, *E. piscisicarius* demonstrated the ability to induce systemic infection and clinical signs in pigs under experimental conditions, even in *Erysipelothrix* spp. vaccinated animals. These findings support its recognition as an emerging swine pathogen and underscore the importance of accurate species-level diagnostics, ongoing surveillance, and evaluation of current vaccine coverage strategies for *Erysipelothrix* spp.

## Figures and Tables

**Table 1:** Liver and spleen wights of pigs from experimental groups (HD, LD, and CONT) at D7 and D14, and their correlation with final body weight.

Macroscopic size alterations in organs mean weight value with standard error of mean (SEM)									
	D7			D14			General		
	Liver (g)	Spleen (g)	Body weight (kg)	Liver (g)	Spleen (g)	Body weight (kg)	Liver (g)	Spleen (g)	Body weight (kg)
HD	900 (79.69)	151 (20.02)	30.4 (2.41)	902 (47.58)	136 (16.91)	31.4 (2.88)	901 (43.75)	143 (12.61)	30.9 (4.1)
LD	938 (41.76)	144 (16.31)	31.6 (4.32)	925 (91.18)	158 (31.21)	30.4 (4.93)	933 (47.31)	151 (16.76)	31 (4.42)
CONT	770 (20)	95 (5)	30.8 (2.48)	990 (150)	105 (35)	32.9 (4.95)	880 (88.60)	100 (14.72)	31.7 (3.4)

**Table 2:** Frequency of histopathological lesions observed in pigs challenged with *E. piscisicarius*.

Group	HD		LD		CONT
Lesion/moment	D7	D14	D7	D14	D14
Pulmonary infiltrate + fibrin^a^	3/5 (60%)	3/5 (60%)	5/5 (100%)	1/5 (20%)	0/6 (0%)
Splenic congestion^a^	4/5 (80%)	5/5 (100%)	0/5 (0%)	2/5 (40%)	0/6 (0%)
Pericarditis (mild)	1/5 (20%)	0/5 (0%)	0/5 (0%)	0/5 (0%)	0/6 (0%)
Tonsillar crypt dilation	0/5 (0%)	1/5 (20%)	0/5 (0%)	1/5 (20%)	0/6 (0%)
Testicular inflammation	1/5 (20%)	0/5 (0%)	0/5 (0%)	0/5 (0%)	0/6 (0%)
Renal mononuclear infiltrate	0/5 (0%)	0/5 (0%)	2/5 (40%)	3/5 (60%)	2/6 (33.3%)
Intestinal desquamation	2/5 (40%)	0/5 (0%)	2/5 (40%)	1/5 (20%)	1/6 (16.6%)
Dermal perivascular infiltrate	0/5 (0%)	0/5 (0%)	3/5 (60%)	1/5 (20%)	2/6 (33.3%)

*Note*: Values represent the number and percentage of affected animals per group.

^a^indicate compatible lesions with swine erysipelas. Lesions attributable to the infection, based on their presence in challenged groups and absence in nonchallenged group.

**Table 3:** Detection of *E. piscisicarius* DNA by qPCR (targeting the *spaC* gene) in blood and tissue samples from challenged HD and LD groups.

DNA detection^a^ in tissue from challenged pigs								
Number of positive/number of tested (%); mean Ct value (SEM)								
Days postchallenge	Blood		Skin		Liver		Splen	
	HD	LD	HD	LD	HD	LD	HD	LD
2	2/10 (20%); 35.7 (1.1)	4/10 (40%); 38.5 (0.84)	NA	NA	NA	NA	NA	NA
7	1/10 (10%); 36.5 (0.0)	0/10 (0%); 0.0 (0.0)	3/5 (60%); 37.8 (1.5)	3/5 (60%); 37.1 (0.46)	3/5 (60%); 37.5 (0.48)	5/5 (100%); 36.3 (1.06)	5/5 (100%); 33.4 (0.25)	5/5 (100%); 33.2 (0.38)
14	0/5 (0%); 0.0 (0.0)	0/10 (0%); 0.0 (0.0)	0/2 (0%); 0.0 (0.0)	2/5 (40%); 38.4 (0.20)	2/5 (40%); 37.1 (0.25)	2/5 (40%); 35.8 (0.45)	5/5 (100%); 34.3 (0.23)	4/5 (80%); 34.3 (0.38)

*Note*: Data shows the number of positives over total samples tested.

Abbreviation: NA, not assessed.

^a^Real-time PCR: samples with Ct (cycle threshold) values <40 was considered positive.

**Table 4:** Serum concentrations (mean ± standard deviation) of acute-phase proteins (APPs) in challenged (HD and LD) and nonchallenged (CONT) pigs after challenge with *E. piscisicarius*.

Groups			
	HD	LD	CONT
Total proteins (g/dL)			
D0 (Challenge)	6.07 (±0.31)^Aa^*⁣*^*∗*^	6.05 (±0.20)^Aa^	6.20 (±0.12)^Aa^
D7	6.20 (±0.18)^Aa^	6.16 (±0.25)^Aa^	5.91 (±0.27)^ABa^
D14	5.88 (±0.11)^Aa^	5.92 (±0.20)^Aa^	5.39 (±0.18)^Ba^
Ceruloplasmin (mg/dL)			
D0 (Challenge)	0.04 (±0)^Aa^	0.03 (±0)^Aab^	0.02 (±0)^Ab^
D7	0.03 (±0.01)^Aa^	0.03 (±0.01)^ABa^	0.05 (±0.01)^Ba^
D14	0.08 (±0.01)^Ba^	0.08 (±0.02)^Ba^	0.06 (±0.01)^Ba^
Transferrin (mg/dL)			
D0 (Challenge)	0.22 (±0.05)^Aa^	0.27 (±0.04)^Aa^	0.26 (±0.03)^Aa^
D7	0.16 (±0.02)^ABa^	0.25 (±0.03)^ABb^	0.40 (±0.02)^Ac^
D14	0.32 (±0.05)^Aa^	0.35 (±0.03)^Aa^	0.34 (±0.04)^Ba^
Albumin (mg/dL)			
D0 (Challenge)	3.89 (±0.40)^Aa^	4.29 (±0.35)^Aa^	4.47 (±0.39)^Aa^
D7	4.00 (±0.38)^Aa^	4.34 (±0.48)^Aa^	3.81 (±0.17)^ABa^
D14	2.93 (±0.25)^Ba^	3.15 (±0.13)^Ba^	2.99 (±0.16)^Ba^
IgA (mg/dL)			
D0 (Challenge)	0.18 (±0.03)^Aa^	0.17 (±0.02)^Aa^	0.13 (±0)^Aa^
D7	0.12 (±0.01)^Aa^	0.14 (±0.04)^Aa^	0.12 (±0.02)^ABa^
D14	0.18 (±0.02)^Aa^	0.18 (±0.01)^Aa^	0.16 (±0.0)^Aa^
α1-antitrypsin (mg/dL)			
D0 (Challenge)	0 (±0)^Aa^	0.05 (±0.07)^Aa^	0.0 (±0)^Aa^
D7	0.04 (±0.07)^Aa^	0.05 (±0.08)^Aa^	0.0 (±0.0)^Aa^
D14	0.14 (±0.07)^Aa^	0.26 (±0.04)^Aa^	0.26 (±0.07)^Aa^
α1-acid glycoprotein (mg/dL)			
D0 (Challenge)	0.003 (±0.001)^Aa^	0.004 (±0.001)^Aa^	0.003 (± 0.001)^Aa^
D7	0.003 (±0.001)^Aa^	0.003 (±0.001)^Aab^	0.006 (± 0)^Ab^
D14	0.005 (±0.002)^Aa^	0.008 (±0.003)^Aa^	0.003 (± 0.001)^Aa^
Haptoglobin (mg/dL)			
D0 (Challenge)	0.03 (±0.01)^Aa^	0.02 (±0.01)^Aa^	0.03 (±0.01)^Aa^
D7	0.04 (±0.01)^Aa^	0.05 (±0.02)^Aa^	0.04 (±0.01)^Aa^
D14	0.03 (±0.02)^Aa^	0.05 (±0.02)^Aa^	0.03 (±0.01)^Aa^
MW 23,000 (mg/dL)			
D0 (Challenge)	0.15 (±0.02)^Aa^	0.12 (±0.01)^Aa^	0.12 (±0.02)^Aa^
D7	0.14 (±0.01)^Aa^	0.13 (±0.02)^Aab^	0.17 (±0)^Aa^
D14	0.15 (±0.02)^Aa^	0.16 (±0.03)^Aa^	0.15 (±0.01)^Aa^
IgG (mg/dL)			
D0 (Challenge)	1.45 (±0.22)^Aa^	0.97 (±0.16)^Ab^	0.93 (±0.11)^Ab^
D7	1.57 (±0.29)^Aa^	1.05 (±0.18)^ABb^	1.12 (±0.08)^Aab^
D14	1.85 (±0.16)^Aa^	1.49 (±0.27)^Bab^	1.20 (±0.10)^Ab^

*⁣* ^*∗*^Values followed by the same lowercase superscript letter in the row and uppercase superscript letter in the column do not differ from each other by Tukey's test (*p*  > 0.05).

## Data Availability

The datasets generated during and/or analyzed in the current study are available from the corresponding author upon reasonable request.

## Supporting Information

Supporting Information Figure S1. Assembly metrics for three isolates, showing similar genome sizes (~1.72–1.79 Mb) and an identical largest contig (263,881 bp); ERY3-R3747/22 used in infection model. Figure S2. Taxonomic identification. All isolates showed >92% genomic similarity to *Erysipelothrix* and clustered with *E. piscisicarius* reference strain. Figure S3. Phylogenetic tree based on WGS of the clinical isolate used in this study. Figure S4. Correlation plots showing associations between qPCR Ct values (inversely related to bacterial load) and physiological or hematological parameters. Table S1. Overview of the vaccination program and health protocols at the swine farm from which the experimental animals were obtained. Table S2. Average nucleotide identity (ANI) analysis between the *E. piscisicarius* reference genome and three field isolates (ERY3, ERY4, and ERY5). All three isolates showed >99.1% identity, confirming species-level classification. Table S3. Summary of rectal temperature (°C) across the experimental period. Values represent means, as well as minimum and maximum temperatures recorded for each group (HD, LD, and CONT). Table S4. Hematological values in pigs experimentally challenged with *E. piscisicarius* at days 0, 7, and 14 post-challenge.
