# Supplementary material for: Pathogenic Potential of Erysipelothrix piscisicarius in Pigs and Its Implications for Surveillance in Brazil
Source: Transbound Emerg Dis. 2025 Sep 5;2025:5618952. doi: 10.1155/tbed/5618952 (PMC12431807; doi:10.1155/tbed/5618952)
Supplement: Supporting Information — Figure S1. Assembly metrics for three isolates, showing similar genome sizes (~1.72–1.79 Mb) and an identical largest contig (263,881 bp); ERY3-R3747/22 used in infection model. Figure S2. Taxonomic identification. All isolates showed >92% genomic similarity to Erysipelothrix and clustered with E. piscisicarius reference strain. Figure S3. Phylogenetic tree based on WGS of the clinical isolate used in this study. Figure S4. Correlation plots showing associations between qPCR Ct values (inversely related to bacterial load) and physiological or hematological parameters. Table S1. Overview of the vaccination program and health protocols at the swine farm from which the experimental animals were obtained. Table S2. Average nucleotide identity (ANI) analysis between the E. piscisicarius reference genome and three field isolates (ERY3, ERY4, and ERY5). All three isolates showed >99.1% identity, confirming species-level classification. Table S3. Summary of rectal temperature (°C) across the experimental period. Values represent means, as well as minimum and maximum temperatures recorded for each group (HD, LD, and CONT). Table S4. Hematological values in pigs experimentally challenged with E. piscisicarius at days 0, 7, and 14 post-challenge. [file 5618952.f1.docx]

**Pathogenic potential of *Erysipelothrix piscisicarius* in pigs and its
implications for surveillance in Brazil**

**Supplementary Material**

1. **Sequencing and bioinformatics**

Next Generation Sequencing (NGS) was performed on the submitted bacterial isolates sample with the desired goal of **“*Erysipelothrix* WGS”**. The sample was sequenced using ***Illumina DNA Prep Kit on MiSeq Micro V2*** 150 paired end reads. The sequence file was analyzed using open-source bioinformatics tools which provide taxonomic classification of the sequencing reads based on Kraken (John's Hopkins University Center for Computation Biology), performs de novo assembly, serotyping, sequence typing, AMR and pangenome analysis.

1. **
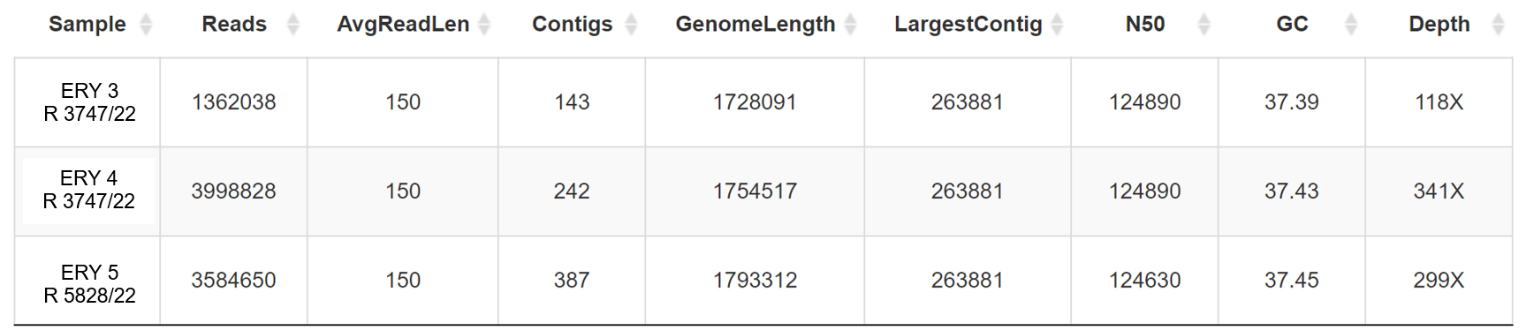
Assembly statistics**

**Figure S1:** Summary of raw read counts, average read length, number of contigs, estimated genome size, largest contig, N50, GC content, and average sequencing depth. All three assemblies shared the same largest contig (263,881 bp), with genome lengths ranging from 1.72 to 1.79 Mb. ERY3-R3747/22 was the isolate used in the experimental infection model.

1. **Taxonomic identification and species confirmation**

**
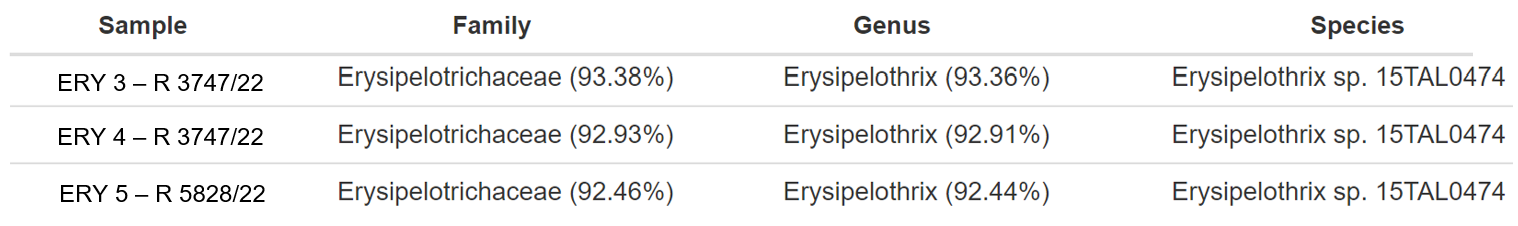
**Taxonomic identification was performed by using Kraken2 (Wood et al., 2019). Further details can be found on the Kraken2 webpage. Additionally, 16S rRNA genes were extracted from each genome by using Barrnap and used for taxonomic classification by using the RDP Classifier (Wang et al., 2007) at a confidence level of 0.8. Further details can be found on the RDPTools webpage.

**Figure S2:** Isolates ERY3-R3747/22, ERY4-R3747/22, and ERY5-R5828/22 was taxonomically classified at the family, genus, and species levels using genome similarity metrics. All isolates showed over 92% nucleotide similarity to the Erysipelothrix genus and were most closely related to the reference strain Erysipelothrix spp. 15TAL0474, supporting their identity as members of the species E. piscisicarius.


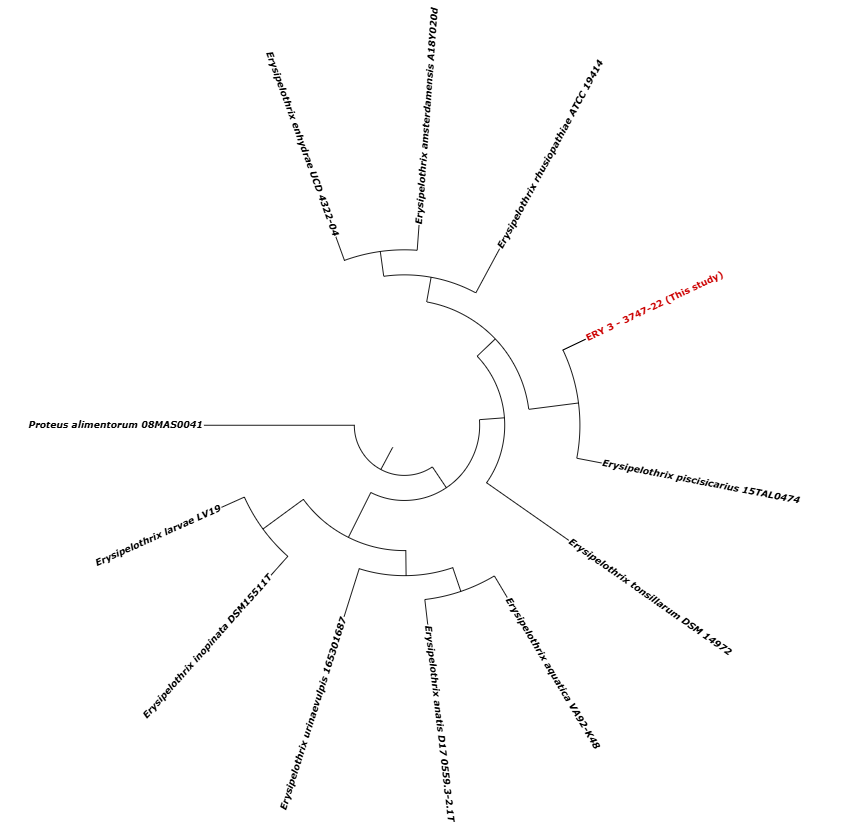


**Figure S3:** Circular phylogenetic tree generated using the TYGS platform. The ERY 3 – 3747/22 isolate (highlighted in red, "This study") clusters closely with Erysipelothrix piscisicarius 15TAL0474, confirming its species identification. The tree was rooted using Proteus alimentorum 08MAS0041 as an outgroup. Tree topology was inferred based on Genome BLAST Distance Phylogeny (GBDP) using whole-genome sequences.


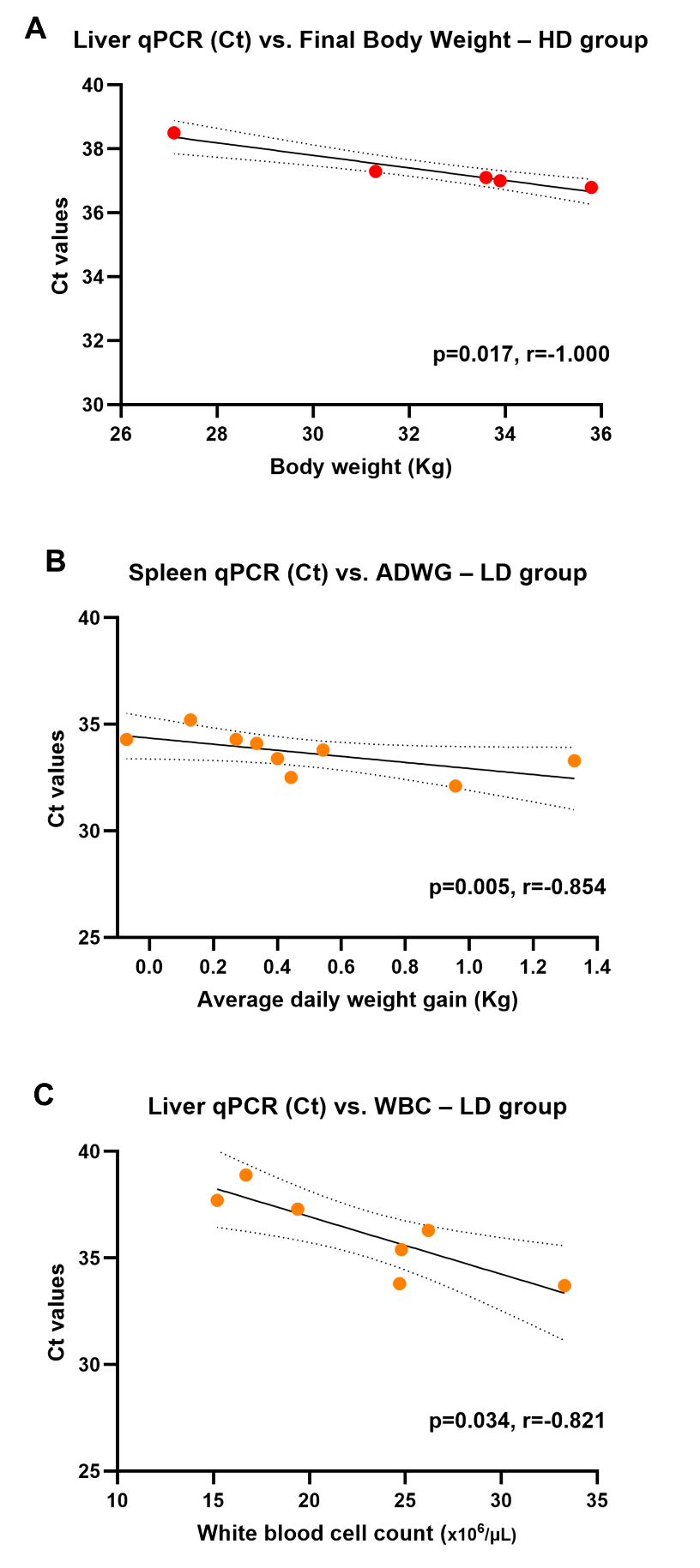


**Figure S4:** Correlation plots showing associations between qPCR Ct values and physiological or hematological parameters. (A) Liver Ct vs. final body weight in the HD group (r = –1.000, p = 0.017); (B) Spleen Ct vs. average daily weight gain in the LD group (r = –0.854, p = 0.005) and (C) Liver Ct vs. white blood cell count (WBC) in the LD group (r = –0.821, p = 0.034). Lower Ct values reflect higher bacterial DNA load. Dotted lines represent 95% confidence intervals.

| **Animal category** | **Vaccines used** |
| --- | --- |
| Weaned piglets | *Circoflex*® (Boehringer Ingelheim, Germany) and *Porcilis Ileitis*® (MSD Animal Health, USA) at weaning (21 days of age). |
| Replacement gilts | *Circoflex*® (Boehringer Ingelheim, Germany), *Suiseng*® (Hipra, Spain), and *Eriseng Parvo Lepto*® (Hipra, Spain) at 175 and 195 days of age. |
| Pregnant sows | *Porcilis*® *Glasser* (MSD Animal Health, USA) and *Suiseng*® (Hipra, Spain) at 75 and 95 days of gestation. |
| Lactating sows | *Eriseng Parvo Lepto*® single dose (Hipra, Spain) and *Circoflex*® (Boehringer Ingelheim, Germany) 7–10 days post-farrowing. |
| Antiparasitic treatment | Mass antiparasitic medication is administered twice per year. |
| General medication | No feed-based medication. Only individual injectable treatments are performed. |

**Table S1:** Vaccination and health management practices at the farm where experimental animals were sourced.

**Table S2:** Average Nucleotide Identity (ANI) analysis between Erysipelothrix piscisicarius reference genome and three field isolates samples (ERY3, ERY4, and ERY5). Pairwise ANI comparisons were performed using OrthoANIu, with E. piscisicarius 15TAL0474 as the reference genome (Genome B). The table displays ANI values (%), total genome lengths of the samples and reference, average aligned sequence lengths, and coverage percentages for both genomes. All field isolates demonstrated >99.1% identity to the reference, confirming species-level similarity.

| **Genome A (Sample ID)** | **Genome B** | **ANI value (%)** | **Genome A length (bp)** | **Genome B length (bp)** | **Average aligned length (bp)** | **Genome A coverage (%)** | **Genome B coverage (%)** |
| --- | --- | --- | --- | --- | --- | --- | --- |
| ERY 3 R 3747/22 | *Erysipelothrix piscisicarius* 15TAL0474 | 99.14 | 1,677,900 | 1,722,780 | 1,046,168 | 62.35 | 60.73 |
| ERY 4 R 3747/22 |  | 99.14 | 1,679,940 | 1,720,780 | 1,063,204 | 63.29 | 61.71 |
| ERY 5 R 5828/22 |  | 99.20 | 1,679,940 | 1,722,780 | 1,137,318 | 67.70 | 66.02 |

**Table S3:** Rectal temperature (°C) means observed during experimental period, followed by the lowest and higher values measured in each group: HD, LD and CONT.

| **Timepoint** | **Group** | **Mean^1*^** | **Min.** | **Max.** |
| --- | --- | --- | --- | --- |
| **D0 (Challenge)** | HD | 38.87^a^ | 38.0 | 39.4 |
|  | LD | 38.79ª | 38.4 | 39.1 |
|  | CONT | 39.13^a^ | 38.8 | 39.4 |
| **D1** | HD | 39.59^a,b^ | 38.2 | 40.7 |
|  | LD | 40.17^a^ | 39.2 | 41.5 |
|  | CONT | 38.58^b^ | 38.4 | 38.8 |
| **D2** | HD | 38.0^a^ | 36.5 | 38.7 |
|  | LD | 38.2^a^ | 36.7 | 39.1 |
|  | CONT | 37.23^a^ | 36.8 | 37.6 |
| **D3** | HD | 37.5^a^ | 36.1 | 38.1 |
|  | LD | 37.23^a^ | 36.3 | 39.1 |
|  | CONT | 37.35^a^ | 37.0 | 37.5 |
| **D4** | HD | 38.45^a^ | 37.5 | 39.5 |
|  | LD | 38.2^a^ | 37.3 | 39.0 |
|  | CONT | 36.75^b^ | 36.4 | 37.1 |
| **D5** | HD | 38.17^a^ | 37.3 | 38.7 |
|  | LD | 38.27^a^ | 37.3 | 39.3 |
|  | CONT | 36.98^b^ | 36.7 | 37.3 |
| **D7** | HD | 38.98^a^ | 38.5 | 39.8 |
|  | LD | 38.92^a,b^ | 37.8 | 40.1 |
|  | CONT | 37.53^b^ | 36.7 | 38.1 |
| **D11** | HD | 38.8^a^ | 38.2 | 39.2 |
|  | LD | 38.82^a^ | 38.0 | 39.3 |
|  | CONT | 37.2^b^ | 36.7 | 37.7 |
| **D14** | HD | 38.56^a^ | 38.2 | 38.9 |
|  | LD | 38.46^a^ | 38.1 | 38.9 |
|  | CONT | 37.33^b^ | 36.7 | 37.8 |
| ^1^Means followed by the same letter on the line do not differ by Tukey test (*p*> 0.05).  * >39.5°C indicates fever according to Dewey and Straw et al (2006). | | | | |

**Table S4:** Hematological parameters in pigs challenged with *E. piscisicarius* at days 0, 7, and 14 post-challenge. Values are presented as mean ± standard deviation. Statistically significant differences (p < 0.05) are indicated between groups (HD, LD and CONT) and across timepoints.

|  | **Groups** | | | |
| --- | --- | --- | --- | --- |
|  | **HD** | **LD** | **CONT** |  |
| **Red blood cell count (x10^6^/μL)** | | | |  |
| **D0**  **(Challenge)** | 6.89(± 0.97)^Aa^ | 6.86(± 0.34)^Aa^ | 6.43(± 1.12)^Aa^ |  |
| **D7** | 5.94(± 1.1)^ABab^ | 5.80(± 0.17)^Aa^ | 7.28(± 0.24)^Ba^ |  |
| **D14** | 4.63(± 0.45)^Ab^ | 4.03(± 1.08)^Ab^ | 5.67(± 0.84)^Aa^ |  |
| **White blood cell count (x10^6^/μL)** | | | |  |
| **D0**  **(Challenge)** | 20.39(± 4.23)^Aa^ | 22.83(± 5.23)^Aab^ | 21.93(± 2.17)^Aa^ |  |
| **D7** | 20.5(± 5.42)^Aa^ | 24.9(± 5.02)^Aa^ | 23.47(± 2.00)^Aa^ |  |
| **D14** | 23.92(± 6.04)^Aa^ | 17.22(± 1.54)^Bb^ | 18.43(± 2.96)^Aba^ |  |
| **Haemoglobin (g/dL)** | | | |  |
| **D0**  **(Challenge)** | 12.41 (± 1.62)^Aa^ | 12.52 (± 1.15)^Aa^ | 11.43 (± 1.77)^Aa^ |  |
| **D7** | 10.5 (± 1.63)^Aba^ | 10.40 (± 0.44)^Aa^ | 12.7 (± 0.44)^Ba^ |  |
| **D14** | 8.0 (± 0.86)^ABb^ | 7.16 (± 1.88)^Aa^ | 11.47 (± 0.57)^Ba^ |  |
| **Haematocrit (%)** | | | |  |
| **D0**  **(Challenge)** | 38.82(± 5.22)^Aa^ | 38.84(± 3.44)^Aa^ | 36.18(± 6.36)^Aa^ |  |
| **D7** | 30.82(± 4.52)^ABab^ | 30.75(± 1.7)^Aab^ | 39.23(± 1.86)^Ba^ |  |
| **D14** | 27.68(± 3.03)^Ab^ | 23.66(± 6.1)^Ab^ | 38.67(± 1.95)^Ba^ |  |
| **MCV (fL)** | | | |  |
| **D0**  **(Challenge)** | 56.35(± 1.35)^Aab^ | 56.65(± 5.12)^Aab^ | 56.45(± 0.84)^Aa^ |  |
| **D7** | 52.22(± 2.92)^Aa^ | 52.96(± 2.13)^Aa^ | 53.91(± 1.41)^Aa^ |  |
| **D14** | 59.67(± 2.42)^Ab^ | 59.08(± 2.67)^Ab^ | 62.37(± 8.22)^Aa^ |  |
| **MCH (pg)** | | | |  |
| **D0**  **(Challenge)** | 18.03(± 0.59)^Aa^ | 18.27(± 1.77)^Aa^ | 17.96(± 0.66)^Aa^ |  |
| **D7** | 17.76(± 0.82)^Aa^ | 17.92(± 0.49)^Aa^ | 17.45(± 0.19)^Aa^ |  |
| **D14** | 17.25(± 0.68)^Aa^ | 17.83(± 0.73)^Aa^ | 20.51(± 2.81)^Aa^ |  |
| **MCHC (g/dL)** | | | |  |
| **D0**  **(Challenge)** | 31.99 (± 0.55)^Aab^ | 32.24 (± 1.12)^Aab^ | 31.71 (± 1.36)^Aa^ |  |
| **D7** | 34.03 (± 0.8)^Aa^ | 33.85 (± 0.92)^Aa^ | 32.38 (± 0.52)^Aa^ |  |
| **D14** | 28.92 (± 1.12)^Ab^ | 30.23 (± 1.98)^Ab^ | 33.10 (± 5.02)^Aa^ |  |
| **Platelet count (x10^3^/μL)** | | | |  |
| **D0**  **(Challenge)** | 510.1 (± 99.93)^Aa^ | 497.7 (± 108.7)^Aa^ | 431.3 (± 92.28)^Aa^ |  |
| **D7** | 406.4 (± 254.6)^Aa^ | 638.0 (± 117.3)^Aa^ | 544.0 (± 64.44)^Aa^ |  |
| **D14** | 365.4 (± 159.8)^Aa^ | 434.4 (± 215.2)^Aa^ | 406.0 (± 14.42)^Aa^ |  |

*Means followed by equal uppercase letters in the same row and equal lowercase letters in the same column did not differ by Tukey's test (*p*>0.05).
